# Classification Rule for 5-year Cardiovascular Diseases Risk using decision tree in Primary Care Chinese Patients with Type 2 Diabetes Mellitus

**DOI:** 10.1038/s41598-017-15579-z

**Published:** 2017-11-10

**Authors:** Eric Yuk Fai Wan, Daniel Yee Tak Fong, Colman Siu Cheung Fung, Esther Yee Tak Yu, Weng Yee Chin, Anca Ka Chun Chan, Cindy Lo Kuen Lam

**Affiliations:** 1 Department of Family Medicine and Primary Care, the University of Hong Kong, 3/F Ap Lei Chau Clinic, 161 Main Street, Ap Lei Chau, Hong Kong, China; 2 School of Nursing, the University of Hong Kong, Hong Kong, China

## Abstract

Cardiovascular disease(CVD) is the leading cause of mortality among patients with type 2 diabetes mellitus(T2DM), and a risk classification model for CVD among primary care diabetic patients is pivotal for risk-based interventions and patient information. This study developed a simple tool for a 5-year CVD risk prediction for primary care Chinese patients with T2DM. A retrospective cohort study was conducted on 137,935 primary care Chinese T2DM patients aged 18–79 years without history of CVD between 1 January 2010 and 31 December 2010. New events of CVD of the cohort over a median follow up of 5 years were extracted from the medical records. A classification rule of 5-year CVD risk was obtained from the derivation cohort and validated in the validation cohort. Significant risk factors included in decision tree were age, gender, smoking status, diagnosis duration, obesity, unsatisfactory control on haemoglobin A1c and cholesterol, albuminuria and stage of chronic kidney disease, which categorized patients into five 5-year CVD risk groups(<5%; 5–9%; 10–14%; 15–19% and ≥20%). Taking the group with the lowest CVD risk, the hazard ratios varied from 1.92(1.77,2.08) to 8.46(7.75,9.24). The present prediction model performed comparable discrimination and better calibration from the plot compared to other current existing models.

## Introduction

Diabetes Mellitus (DM) is a leading cause of global burden affecting 415 million people in the world and expected to increase to 642 million by 2040^1^. The estimated health expending on diabetes continues to rise to US$673 billion, accounting for 11.6% of global health expenditure^1^. The medical costs for diabetic patients with cardiovascular diseases (CVD) is at least two-fold higher than those without CVD^2^, and constitutes 70% of mortality^3^. Numerous studies have found that CVD events can be prevented or delayed with lifestyle modification or use of pharmacotherapy in diabetic patients^4,5^. An important step in preventing or delaying the incidence of CVD is to identify diabetic patients who are at high risk so that they can receive earlier or more intensive intervention.

Most guidelines recommend that risk-stratified interventions for the management of DM in primary care should be delivered based on predicted overall CVD risk profile rather than individual risk factors and predicted subtype of CVD risk such as stroke and coronary heart disease^4,5^. Patients classified as low risk may be monitored less frequently, whereas patients classified as high risk may be given a closer and more intensive monitoring and intervention. Several CVD risk models have been developed to estimate the risk for stratifying patients into specific risk categories, in order to facilitate clinicians in making medical decisions or determining the effects of an intervention. Most models usually include individual prognostic factors but ignore their interaction effects even though studies have shown interaction effects with age^6,7^, and gender^8,9^. Furthermore, most of models have been developed and evaluated on predominantly Caucasian populations which may not be applicable to Chinese populations. Chinese account for approximately 25% of the diabetic population worldwide^1^, and a multinational study from the World Health Organization showed an ethnic disparities in CVD prevalence among diabetic population^10,11^. Hence, there is a need to derive a Chinese specific risk classification model that accounts for potential interaction effects for CVD incidence. The aim of this study was to develop a simple risk classification tool to fast determine the 5-year CVD risk for patients with Type 2 DM (T2DM) which could be readily implemented into routine clinical use using readily available clinical data.

## Results and Discussion

A total of 137,935 eligible Chinese patients, aged between 18 and 79 years, were diagnosed clinically with T2DM and managed in public primary care clinics and without past history of CVD or end stage renal disease. Random split of the dataset resulted in 91,956 and 45,979 subjects in the derivation and validation cohorts, respectively. During the median follow-up period of 5.0 years in both cohorts, the number of CVD events in the derivation and validation cohorts were 8,124 and 4,154, respectively. Table 1 shows the baseline characteristics between the two cohorts. In general, there were more females (53.3%) than males (46.7%); the mean age was 62.1 years (standard deviation (SD): 10.0 years); 11.2% were current smokers and the mean duration of T2DM was 7.0 years (SD: 6.2 years).

**Table 1:** Baseline characteristics of derivation and validation cohorts.

	Derivation Cohort (N = 91,956)	Validation Cohort (N = 45,979)	p-value
**Socio-Demographics**			
Gender			0.966
Female	53.3% (48,982)	53.3% (24,486)	
Male	46.7% (42,974)	46.7% (21,493)	
Age,years	62.06 ± 9.98 (91,956)	61.95 ± 9.98 (45,979)	0.065
<50 years	11.0% (10,146)	11.0% (5,050)	0.226
50–64 years	47.2% (43,400)	47.7% (21,923)	
≥65 years	41.8% (38,410)	41.3% (19,006)	
Smoking Status			0.765
Non-Smoker	88.8% (81,169)	88.8% (40,595)	
Smoker	11.2% (10,247)	11.2% (5,097)	
**Clinical Characteristics**			
Duration of T2DM, years	7.00 ± 6.23 (85,063)	7.02 ± 6.26 (42,529)	0.516
<2 years	18.0% (15,307)	18.0% (7,656)	0.534
2–5 years	25.1% (21,345)	24.8% (10,553)	
≥5 years	56.9% (48,411)	57.2% (24,320)	
Diabetic Retinopathy			0.870
No	50.6% (46,554)	50.7% (23,299)	
Yes	49.4% (45,402)	49.3% (22,680)	
Obesity			0.966
No	31.6% (23,994)	31.6% (11,975)	
Yes	68.4% (51,957)	68.4% (25,946)	
Unsatisfactory control on HbA1c			
No	80.4% (72,606)	80.1% (36,169)	0.205
Yes	19.6% (17,691)	19.9% (8,976)	
Unsatisfactory control on BP			
No	61.4% (56,299)	61.7% (28,251)	0.379
Yes	38.6% (35,356)	38.3% (17,559)	
Unsatisfactory control on cholesterol			
No	60.1% (52,781)	60.1% (26,386)	0.947
Yes	39.9% (35,054)	39.9% (17,510)	
Albuminuria	l		
No	74.2% (48,422)	74.1% (24,207)	0.803
Microalbuminuria	21.3% (13,930)	21.3% (6,962)	
Macroalbuminuria	4.5% (2,923)	4.6% (1,493)	
Stage of chronic kidney disease			
Stage 1	73.1% (65,492)	73.2% (32,714)	0.892
Stage 2	23.0% (20,646)	23.0% (10,298)	
Stage 3 or above	3.9% (3,461)	3.8% (1,704)	

HbA1c = Haemoglobin A1c; BP = Blood pressure; T2DM = Type 2 diabetes mellitus; Notes: *Significant difference at 0.05 level by chi-square test or independent t-test, as appropriate.

**Figure 1:**
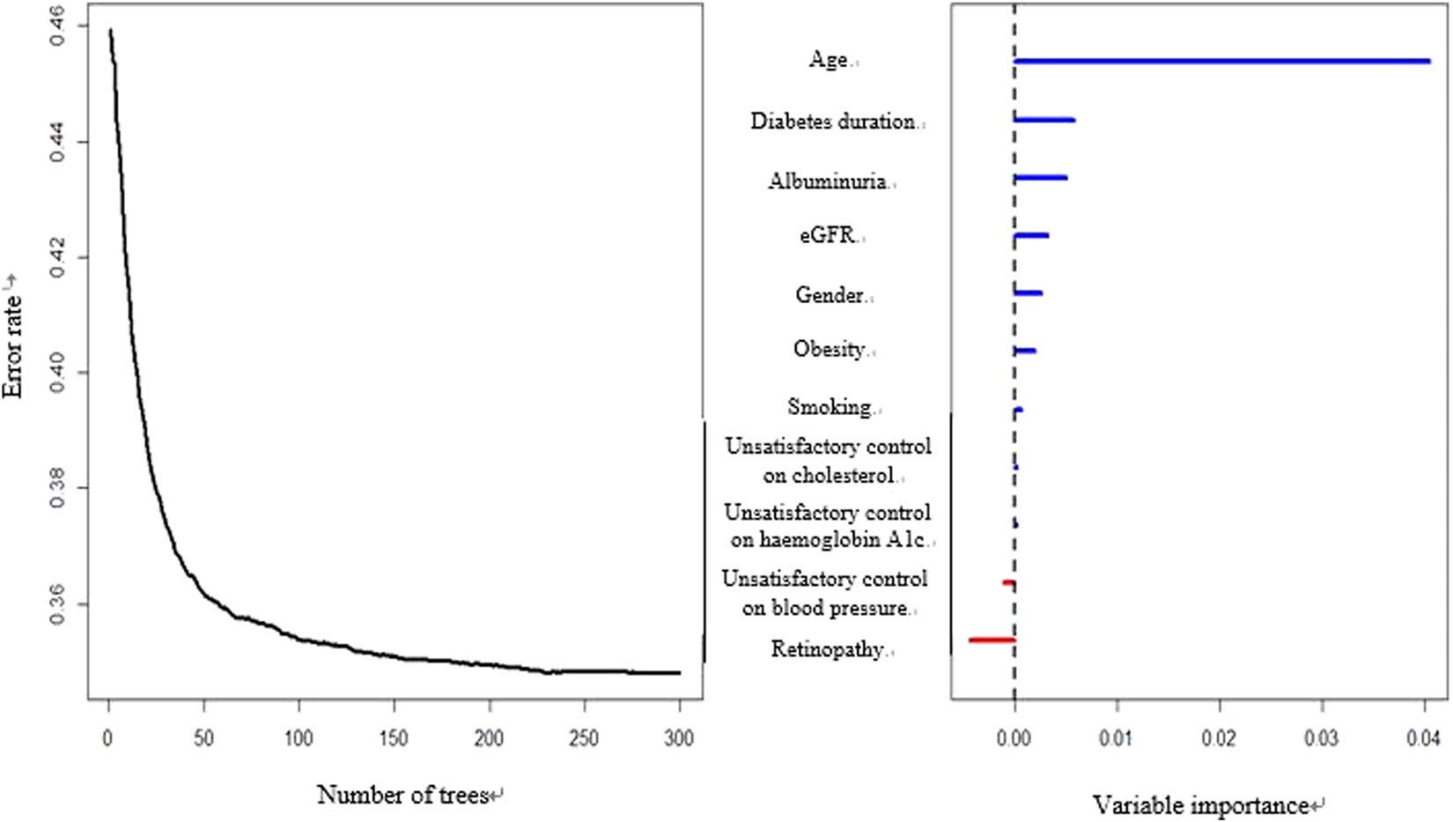
Error rate and variable importance from the random survival tree analysis using the *rfsrc* function with 300 trees from the *randomForestSRC* package in R for the cardiovascular diseases event.

**Figure 2:**
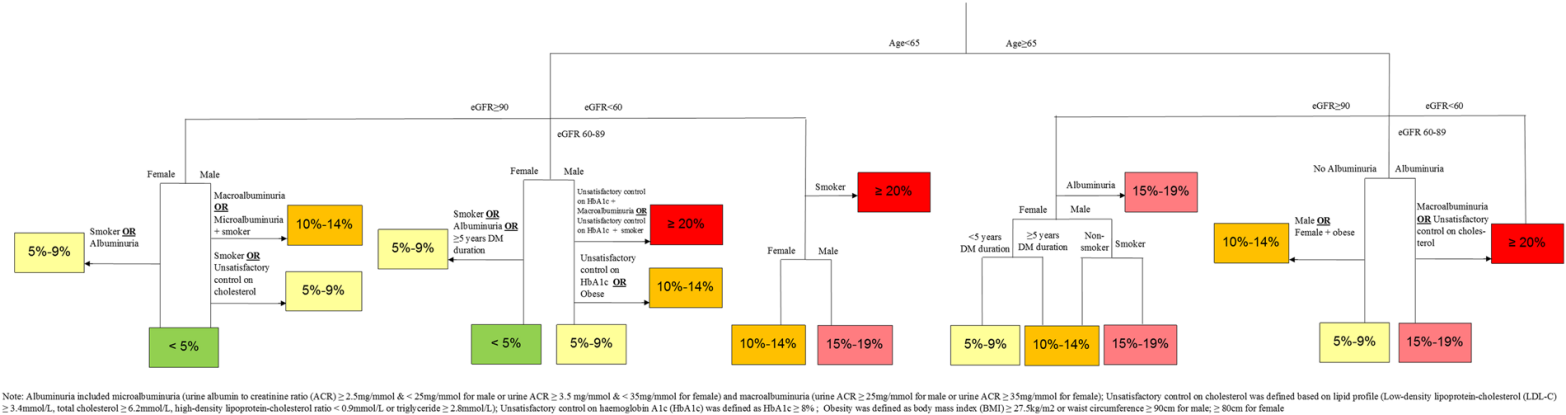
Simplified survival tree on 5-year cardiovascular disease risk.

**Figure 3:**
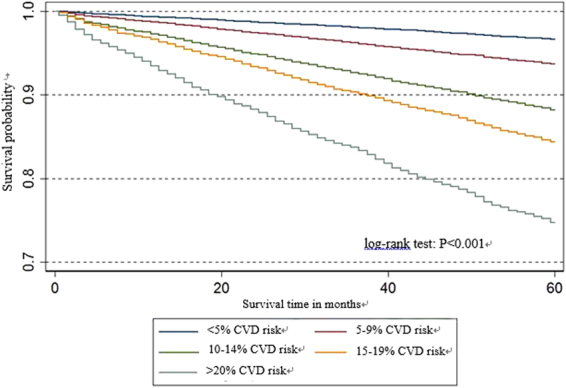
Kaplan-Meier survival curves of the five final risk groups.

Figure 1 shows the variable importance of all potential predictors. Unsatisfactory control on BP and diabetic retinopathy were excluded from main analysis since they had negative importance scores. A survival tree model was fitted using the remaining potential predictors with positive importance scores including age, gender, smoking status, diabetes duration, obesity, unsatisfactory control on HbA1c and cholesterol, albuminuria and estimated glomerular filtration rate (eGFR), and 38 terminal nodes/groups were obtained and shown in Supplementary Figure 1. The average absolute 5-year CVD risk among these groups varied from 1.73% to 39.6%, and hazard ratios (HR) by taking the group with the lowest CVD risk as reference level were between 1.8 and 28.9. After stratifying each terminal node/group into one of the five different severity risk groups (<5%; 5–9%; 10–14%; 15–19% and ≥20%), a final simplified tree was constructed in Figure 2. Figure 3 showed the Kaplan-Meier curves for these five groups and showed significant difference between groups by log-rank test. Taking the group with the lowest CVD risk, the hazard ratios for other groups varied from 1.92 (1.77, 2.08) to 8.46 (7.75, 9.24) in Table 2. All pairwise comparisons were statistically significant, indicating the stratification of the CVD risk was effective and appropriate to separate the subjects.

**Table 2:** Hazard ratios for the five final risk groups of cardiovascular diseases.

CVD risk group	HR (95% CI)	p-value for testing the pairwise difference*			
		Low	Very low	Medium	High
<5%	Reference group				
5–9%	1.92 (1.77,2.08)	<0.001			
10–14%	3.67 (3.40,3.96)	<0.001	<0.001		
15–19%	4.95 (4.56,5.37)	<0.001	<0.001	<0.001	
≥20%	8.46 (7.75,9.24)	<0.001	<0.001	<0.001	<0.001

HR = Hazard Ratio; CI = Confidence Interval. Notes: *All pairwise comparisons are statistically significant after adjusting for multiplicity using Holm’s procedure.

**Figure 4:**
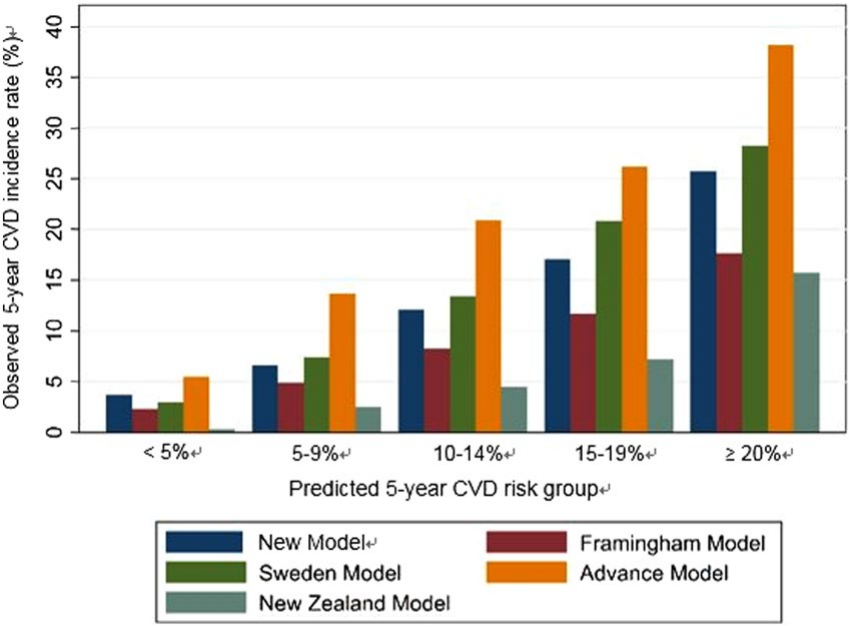
Calibration plots for observed and predicted 5-year risks of cardiovascular disease.

The performance of the new model was compared with existing CVD risk models using the validation cohort and the results are shown in Table 3. In terms of predictive power, the Harrell’s C statistic for new model was 0.677, which was similar with Swedish model (0.685) and was superior than the others and the difference also reached statistically significance. Supplementary Table 1 showed similar results using 10-fold cross validation. Meanwhile, the calibration power of the five different models on five different CVD risk groups was compared in Figure 4. The five predicted 5-year CVD risk groups were matched with the observed CVD risk for our model, indicating a good calibration. Swedish model slightly underestimated the observed 5-year CVD incidence rate in the predicted 15–20% risk group. Other models showed a poor calibration with either an underestimation or an overestimation on the actual CVD risk.

**Table 3:** Performance of new and existing CVD risk models in validation cohort for predicting 5-year risk of cardiovascular disease.

Validation statistics	New Model	Framingham	Swedish model	ADVANCE	New Zealand model
Harrell’s C statistic	0.677 (0.669,0.685)	0.660 (0.652,0.667)	0.685 (0.677,0.693)	0.665 (0.657,0.673)	0.671 (0.664,0.678)
D statistic	1.054 (1.003,1.112)	0.983 (0.927,1.040)	1.128 (1.079,1.187)	1.104 (1.051,1.153)	1.278 (1.214,1.352)
R^2^	0.228 (0.207,0.248)	0.182 (0.166,0.199)	0.250 (0.230,0.270)	0.223 (0.205,0.243)	0.225 (0.208,0.246)
Brier score	0.079 (0.077,0.081)	0.080 (0.078,0.082)	0.079 (0.077,0.081)	0.079 (0.077,0.081)	0.080 (0.077,0.082)

Notes: The brackets represent 95% confidence interval of corresponding validation statistic. *Significant difference in Harrell’s C statistic (P-value < 0.05).

This study developed a simple risk stratification tool to quickly establish the CVD risk for patients with DM using clinical information extracted from a territory-wide patient dataset. Our developed model had comparable discriminatory and superior calibration power when compared to other developed CVD prediction models. The current findings also identified the importance of the presence of chronic kidney disease and albuminuria in predicting CVD risk amongst Chinese patients with DM.

This is the first study to use a decision tree analysis to identify and classify CVD risk in patients with T2DM. The main advantage of using a decision tree analysis is the ability to convert complicated risk equations into an organized flowchart, which can be easily navigated to identify the appropriate risk. This is important in clinical practice, where short consultation times can make more complex risk stratification tools less amenable to being used. A simple, practical and user-friendly approach can help promote clinicians to make more valid risk-based decisions on intervention. Furthermore, this tool can help to minimize the need for unnecessary measurements with a view to reduce the treatment burden and unnecessary use of health resources.

There were nine predictors in our developed model, which is lower than other existing models based on diabetic populations^12–14^. Furthermore, other models usually rely on a single sophisticated mathematical formula, which may not work if one of parameters is missing. In our developed model, it is still possible to estimate a risk level with missing data. For example, in a male, non-smoker, aged <65 years old, with eGFR ≥90 ml/min/1.73 m^2^ and no albuminuria but missing lipid profile, this patient can be classified into either <5% or 5–9% risk group, which indicates the 5-year CVD risk should be less than 10%.

Regarding the accuracy of the risk prediction, our model demonstrated similar discrimination but better calibration power than other existing models. Calibration, which measures the agreement between observed and predicted risks, is an immense indicator for assessment of the performance of the model. Currently, several international guidelines on diabetes management take CVD risk stratification into account and thus a lack of calibration power, either underestimated or overestimated risk of CVD, may result in improper risk-based interventions. An apparent disparity between observed and predicted risks by other existing models developed based on predominantly Caucasian population may possibly be attributed to ethnic discrepancy. Several multi-ethnic epidemiological studies including the World Health Organization showed that the prevalence of CVD was doubled in Caucasians compared to Chinese^10,11^, and even the risk of CVD among Asian diabetic population was vastly different between Malay, Asian Indian and Chinese^15^. The reasons for different ethnic CVD risks are thought to be due in part to differences in disease profile, genetics and cultural lifestyles^10,11,16–18^, and thus the prediction model for CVD should be ideally be ethnic-specific.

One key feature in our developed model was the inclusion of both eGFR and urine albumin/creatinine ratio (ACR) as indicators of the severity of renal impairment. A recent meta-analysis with over 600,000 patients from 24 cohorts conducted by the Chronic Kidney Disease Prognosis Consortium (CKD-PC) also found that urine ACR and eGFR were independent risk factors for CVD, and suggested to incorporate both for CVD risk prediction^19^. Our analyses also revealed the ranking importance for ACR and eGFR were at the third and fourth levels, which were higher than other traditional predictors such as lipid profile. The pathophysiological pathways between renal impairment and CVD risk are still not completely understood^20^. In general, diabetes is one of major prognostic factors for the progression of atherosclerosis and accelerated atherosclerosis is observed in severe chronic kidney disease resulting in increased risks for CVD^20,21^. Indeed, there is much concern on the enormously detrimental effect of diabetic kidney diseases in CVD risk in the world, particular in Chinese populations^22^. Multinational studies have found that Chinese have higher risks of renal impairment than non-Chinese^10,11,22–24^. Hence, the simultaneous assessment of eGFR and ACR appears to be of key importance in deriving CVD risk.

There were several strengths to this study. The dataset used to develop the model was derived from the computerised database of the Hong Kong Hospital Authority (HA), and is representative of the Chinese diabetic population managed in primary care setting. The clinical and laboratory data were reliable and accurate as they were and extracted systemically directly from the HA’s administrative database.

There were also several limitations. First, our study design was a retrospective one, which may produce biased results when compared with a prospective study. Second, other potential predictors such as exercise, diet and diabetes education level were not captured in our database and may be worthy of consideration in future studies. Third, the data for this study was derived from patients receiving diabetes care in primary care, and the model may not be valid in patients being managed in secondary or tertiary care settings. Studies with a longer follow-up period, are still needed to estimate the 10-year or longer CVD risk. Further studies to assess its validity and reliability in other settings are needed to examine the performance of the model in other Chinese populations.

## Conclusion

Our developed model using tree analysis techniques is able to provide accurate and evidence-based CVD risk predictions for Chinese diabetic patients. This can be translated into a decision making tool to help inform clinicians regarding intervention choice based on predicted risk rather than relying on a single risk factor. The predicted risk stratification can educate, motivate and empower patients to prevent future DM complications. At the health policy level, the risk distribution can inform decisions on better service provision and resource allocation. For researchers, the prediction models can be used as an indicator to measure the potential benefits of complication prevention in clinical trials on DM interventions in primary care. Further studies are needed with a longer follow-up period o to estimate long-term CVD risks and to validate the model in other Chinese populations.

## Subjects, Materials and Methods

### Study Design

This population-based retrospective cohort study included Chinese patients aged between 18 and 79 years, were clinically diagnosed with T2DM, managed in public primary care clinics and without past history of CVD or end stage renal disease. Clinical data were collected and retrieved through the administrative database of the HA for patients who had received primary care services from one of the 74 general out-patient clinics of the HA between 1 January 2010 and 31 December 2010. The HA is the centralized governmental organisation who governs all public-sector hospitals and primary care clinics, and manages over 50% of DM patients under primary care in Hong Kong. Data were made available as part of a territory-wide study on the evaluation of the quality of care of a government-subsidized primary care DM management program^25^. The clinical diagnosis of T2DM was identified by the International Classification of Primary Care-2 (ICPC-2) code of ‘T90’. The presence of CVD including ischaemic heart disease, myocardial infarction, coronary death and sudden death, heart failure and fatal and non-fatal stroke was identified by the ICPC-2 of ‘K74’ to ‘K77’ and ‘K89’ to ‘K91’, or International Classification of Diseases, Ninth Edition, Clinical Modification (ICD-9-CM) of 410.x to 414.x, 428.x, 430.x to 438.x and 798.x. The date of the earliest attendance records was defined as baseline. Each patient was followed-up until the date of diagnosis of a CVD event, death or the last follow-up as per the censoring date of 30 November 2015, whichever occurred first.

Consent of participants was not necessary as all data were anonymous and were extracted through the computerized administrative system of the Hospital Authority. Ethics approval was received from all the regional Institutional Review Boards (IRB) of the Hong Kong Hospital Authority. The reported investigations have been carried out in accordance with the principles of the Declaration of Helsinki as revised in 2008.

### Potential Classifiers

Potential classifiers consisted of patient’s socio-demographics and clinical characteristics. Socio-demographics included gender, age (<50; 50–64 and ≥65 years old) and smoking status (non-smoker and smoker). Clinical characteristics consisted of self-reported duration of T2DM (<2; 2–5 and ≥5 years) and presence of diabetic retinopathy. Clinical parameters were categorized based on the local framework for diabetes management^26^. Obesity was defined as body mass index (BMI) ≥27.5 kg/m^2^ or waist circumference ≥90 cm for male; ≥80 cm for female; Unsatisfactory control on haemoglobin A1c (HbA1c) was defined as HbA1c ≥8%; Unsatisfactory control on blood pressure (BP) was defined as systolic blood pressure (SBP) ≥140 mmHg or diastolic blood pressure (DBP) ≥90 mmHg; Unsatisfactory control on cholesterol was defined based on lipid profile (Low-density lipoprotein-cholesterol (LDL-C) ≥3.4 mmol/L, total cholesterol ≥6.2 mmol/L, high-density lipoprotein-cholesterol ratio <0.9 mmol/L or triglyceride ≥2.8 mmol/L). Albuminuria included microalbuminuria (urine ACR ≥2.5 mg/mmol & <25 mg/mmol for male or urine ACR ≥3.5 mg/mmol & <35 mg/mmol for female) and macroalbuminuria (urine ACR ≥25 mg/mmol for male or urine ACR ≥35 mg/mmol for female). The severity of kidney disease was stratified into 3 levels (eGFR ≥90 ml/min/1.73 m^2^; 60–89 ml/min/1.73 m^2^ and <60 ml/min/1.73 m^2^). All laboratory assays were performed in accredited laboratories by the College of American Pathologists, the Hong Kong Accreditation Service or the National Association of Testing Authorities, Australia.

### Data Analysis

The cohort was randomly split on a 2:1 basis, in which two-third of patients were taken as the derivation cohort for establishing the classification rule for 5-year CVD risk and the remaining one-third forming the validation cohort for validation of the developed rule. Descriptive statistics (percentages for categorical variables, and means and standard deviations for continuous variables) of baseline characteristics were displayed for both cohorts.

Based on the deviation cohort, we ranked potential classifiers based on their permutation importance by a random survival forest analysis using the *randomForestSRC* package in R and those classifiers with negative importance scores were excluded from further analysis^27^. Then, we constructed a survival tree model by binary recursive partitioning of potential predictors that maximized between-group difference and allowed their interactions, using the *party* package in R^28^. The survival tree was constructed in a conditional inference framework, which ensured that the right sized tree was developed and required no form of pruning or cross-validation^29^. The HR and average absolute risk of each group, defined by the terminal nodes of the tree, was estimated by Cox proportional hazards regression model. For the purpose of fast CVD risk classification in the clinical practice, the groups were combined into five risk groups (<5%; 5–9%; 10–14%; 15–19% and ≥20%). The hazard ratio for each final risk group was estimated and log-rank test was conducted to compare the incidence of CVD between final risk groups.

Using the validation cohort, the performance of the risk classification rule was compared with the ADVANCE, Swedish and New Zealand CVD risk scores for T2DM and Framingham CVD risk score for the general population^12–14,30^. Estimated risks from these models were categorized into five risk groups. The Harrell’s C statistic, D statistic, R^2^ statistic and Brier score were calculated for each model to assess the predictive power of each model. The Harrell’s C statistic is similar to the area under the curve after taking into account the censoring pattern of the patients. A Harrell’s C statistic higher than 0.7 suggests good discrimination of the models^31^. The D statistic is a measure of discrimination where higher value indicates better discrimination. The R^2^ statistic is a measure of explained variation with a higher value indicating better performance. The Brier score is also a measure of discrimination where a lower value indicates greater accuracy. The corresponding 95% confidence intervals (CIs) were obtained by bootstrapping of size 500. Same analyses were repeated by 10-fold cross validation. Calibration plots were generated based on the 5 risk groups and observed CVD risks that were obtained by 5-year Kaplan-Meier estimates in order to evaluate the agreement between predicted and observed CVD risks.

All significance tests were two-tailed and those with p-values less than 0.05 were considered statistically significant. The statistical analysis was performed in STATA Version 13.0 and R Version 3.3.1.

### Data Availability

The data that support the findings of this study are available from the HA but restrictions apply to the availability of these data, which were used under license for the current study, and so are not publicly available. Data are however available from the authors upon reasonable request and with permission of the HA.

## Electronic supplementary material

Supplementary Material
