# Supplementary material for: Classification Rule for 5-year Cardiovascular Diseases Risk using decision tree in Primary Care Chinese Patients with Type 2 Diabetes Mellitus
Source: Sci Rep. 2017 Nov 10;7:15238. doi: 10.1038/s41598-017-15579-z (PMC5681694; doi:10.1038/s41598-017-15579-z)

Classification Rule for 5-year Cardiovascular Diseases Risk using decision tree in Primary Care Chinese Patients with Type 2 Diabetes Mellitus

Authors: Eric Yuk Fai Wan[1], Daniel Yee Tak Fong[2], Colman Siu Cheung Fung[1], Esther Yee Tak Yu[1], Weng Yee Chin[1], Anca Ka Chun Chan[1], Cindy Lo Kuen Lam[1]

[1] Department of Family Medicine and Primary Care, the University of Hong Kong, 3/F Ap Lei Chau Clinic, 161 Main Street, Ap Lei Chau, Hong Kong.

[2] School of Nursing, the University of Hong Kong, Hong Kong

Supplementary Materials

Supplementary Figure 1. Regression tree generated for eligible subjects

Supplementary Table 1. Performance of new and existing CVD risk models in validation cohort for predicting 5-year risk of cardiovascular disease by 10-fold cross validation

Supplementary Table 1. Performance of new and existing CVD risk models in validation cohort for predicting 5-year risk of cardiovascular disease by 10-fold cross validation

Validation statistics	New Model	Framingham	Swedish model	ADVANCE	New Zealand model
Harrell's C statistic	0.677 (0.651,0.704)	0.660 (0.634,0.686)	0.686 (0.660,0.711)	0.665 (0.639,0.691)	0.670 (0.648,0.693)
D statistic	1.057 (0.899,1.215)	0.987 (0.817,1.157)	1.132 (0.969,1.295)	1.105 (0.950,1.260)	1.281 (1.063,1.500)
R^2	0.229 (0.169,0.297)	0.184 (0.130,0.242)	0.252 (0.190,0.322)	0.224 (0.161,0.295)	0.226 (0.169,0.287)
Brier score	0.079 (0.073,0.085)	0.080 (0.073,0.086)	0.079 (0.072,0.085)	0.079 (0.073,0.085)	0.079 (0.073,0.086)

Notes:

The brackets represent 95% confidence interval of corresponding validation statistic

* Significant difference in Harrell's C statistic (P-value < 0.05)

Figure 1. Regression tree generated for eligible subjects

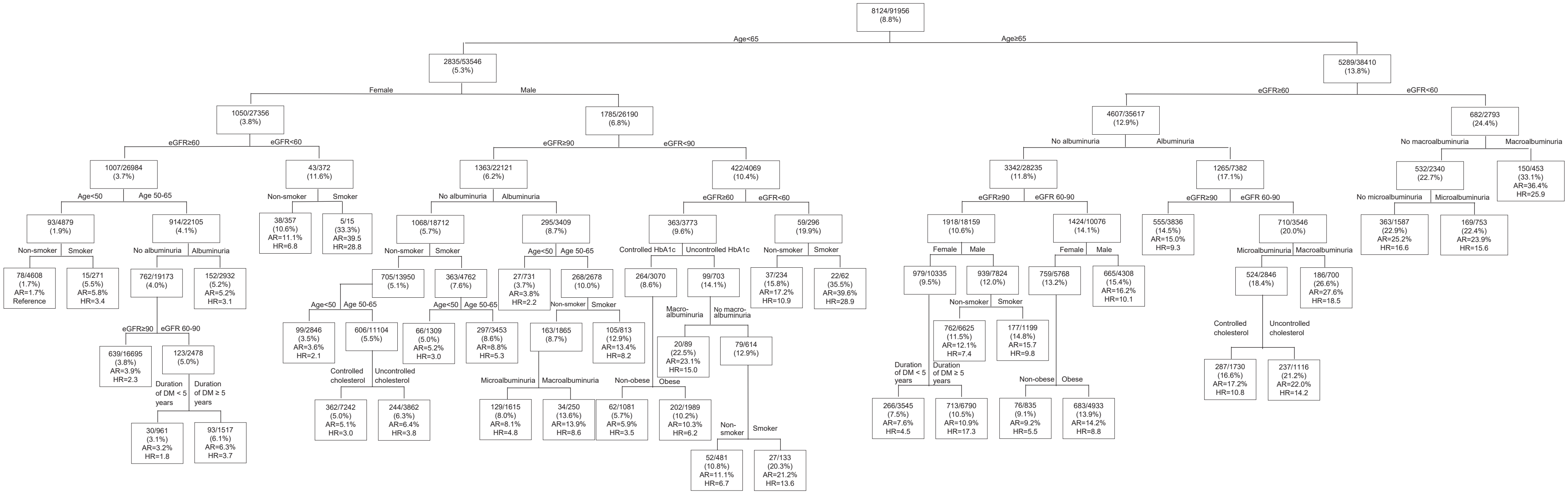

Supplement: Supplementary file 1 — Supplementary Material [file 41598_2017_15579_MOESM1_ESM.pdf]
